# Notes and Notices

**Published:** 1899-11

**Authors:** 


### NOTES AND NOTICES.

### BLOOD CURE OF CHRONIC CATARRH.

CASE OF DR. T. J. B., NEW YORK.

Ethtel Strobel; age 29; May 3d, 1897; naso-pharyngeal catarrh of long standing; had been treated by various specialists, at many clinics; the passages covered with hard crusts, with points of ulceration; severe headache almost constant.

Treatment as already described, and spray of bovinine and salt water every two hours. In ten days the abnormal secretion almost ceased, and the constant catarrhal headache had entirely disappeared. When last seen, June 14th, the ulcerations were entirely healed, and the passages looked healthy.

### CHRONIC ULCERATION NASO-PHARYNGEAL CATARRH.

BY DR. T. J. BIGGS, SOUND VIEW HOSPITAL.

Sam Ellard, New Haven, Conn.; age 26; June 10th, 1898. Had undergone four operations; under the care of the best specialists, for eight years; growing steadily worse. Anterior nares, entire left, one ulcer; six ulcers in the right. Passages now depurated with peroxide-on-bovinine, and touched up with Paquelin cautery. Both nasal passages daily depurated, packed with bovinine in gauze and sprayed with bovinine every two hours. Bovinine also given internally, as in all cases. July 9th, the healing was complete, all over; a result without a parallel to my knowledge.

### Valuable in Changeable Weather.

People are benefited by the use of Speer's Port Grape Wine, especially ladies. It purifies the blood and makes their eyes shine like stars.

### OLD ULCERATIVE CATARRH.

### Case of Dr. R——, New York.

Michael Navarro, age 27; August 12th, 1897; case of many years' standing, treated at various institutions without result; ulceration of the surfaces exuding such large quantities of mucus as to cause severe attacks of vomiting, followed by hemorrhage. After thoroughly cleansing, I sprayed bovinine and salt water into the passages every two hours. Within a week many small points of ulceration had healed, and others were rapidly becoming covered. September 10th, examination showed complete healing of all the ulcers.

### Females and Weakly Persons

At this season should use Speer's Port Grape Wine. Physicians recommend it as a strengthening and blood-purifying tonic, and the best wine to be obtained.

Death Followed Vaccination.—Frank Swalsmy, aged eight years, of Spring Garden, near Mt. Pleasant, died Oct. 23d, from the effects of vaccination. Owing to the small-pox scare in that locality, the boy was vaccinated a week ago by a physician. Five days thereafter, the boy became very ill, his arm swelled and broke out in pimples resembling small-pox. He suffered terribly until death came.

See the girls in another column carrying large baskets of grapes to a winery in Portugal for making wine. It is worth reading about. Speer, of New Jersey, makes wine from the same grape. His wines are unsurpassed by any in the world.

